# Structure-based pharmacophore modeling, virtual screening, and molecular dynamics simulation studies for identification of *Plasmodium falciparum* 5-aminolevulinate synthase inhibitors

**DOI:** 10.3389/fmed.2022.1022429

**Published:** 2023-01-12

**Authors:** Gbolahan O. Oduselu, Rufus Afolabi, Ibitayo Ademuwagun, Ashley Vaughan, Ezekiel Adebiyi

**Affiliations:** ^1^Covenant University Bioinformatics Research (CUBRe), Covenant University, Ota, Ogun State, Nigeria; ^2^Department of Chemistry, Covenant University, Ota, Ogun State, Nigeria; ^3^Department of Biochemistry, Covenant University, Ota, Ogun State, Nigeria; ^4^Center for Global Infectious Disease Research, Seattle Children’s Research Institute, Seattle, WA, United States; ^5^Department of Computer and Information Science, Covenant University, Ota, Ogun State, Nigeria; ^6^Covenant Applied Informatics and Communications ACE (CApIC-ACE), Covenant University, Ota, Ogun State, Nigeria; ^7^Division of Applied Bioinformatics, German Cancer Research Center (DKFZ), Heidelberg, Germany

**Keywords:** 5-ALAS, drug design, heterocycles, malaria, pharmacophore modeling, structure assessment

## Abstract

*Plasmodium falciparum* (*Pf*) 5-aminolevulinic acid synthase (5-ALAS) is an essential enzyme with high selectivity during liver stage development, signifying its potential as a prophylactic antimalarial drug target. The aim of this study was to identify important potential lead compounds which can serve as inhibitors of *Pf* 5-ALAS using pharmacophore modeling, virtual screening, qualitative structural assessment, *in silico* ADMET (Absorption, Distribution, Metabolism, Excretion and Toxicity) evaluation and molecular dynamics simulation. The best model of the tertiary structure of *Pf* 5-ALAS was obtained using MolProbity, while the following databases were explored for the pharmacophore-based virtual screening: CHEMBL, ChemDiv, ChemSpace, MCULE, MCULE-ULTIMATE, MolPort, NCI Open Chemical Repository, LabNetwork and ZINC databases. 2,621 compounds were screened against the modeled *Pf* 5-ALAS using AutoDock vina. The post-screening analysis was carried out using Discovery Studio while molecular dynamics simulation was performed on the best hits using NAMD-VMD and Galaxy Europe platform. Compound **CSMS00081585868** was observed as the best hit with a binding affinity of -9.9 kcal/mol and predicted Ki of 52.10 nM, engaging in seven hydrogen bonds with the target’s active site amino acid residues. The *in silico* ADMET prediction showed that all ten best hits possessed relatively good pharmacokinetic properties. The qualitative structural assessment of the best hit, **CSMS00081585868**, revealed that the presence of two pyridine scaffolds bearing hydroxy and fluorine groups linked by a pyrrolidine scaffold contributed significantly to its ability to have a strong binding affinity with the receptor. The best hit also showed stability in the active site of *Pf* 5-ALAS as confirmed from the RMSD obtained during the MD simulation.

## 1. Introduction

Malaria, a disease caused by eukaryotic *Plasmodium* parasites, continues to kill upward of 6,27,000 people per year and with a further 241 million clinical cases (1). Parasite resistance to antimalarial drugs continues to pose a threat to malaria prevention and control, placing an increasing responsibility on the development of alternative therapies (2). Amongst the metabolic processes critical to *Plasmodium falciparum* (*Pf*) growth during the liver stage of development is the heme biosynthetic pathway (3–5). The rate-limiting enzyme in heme biosynthesis, 5-aminolevulinic acid synthase (5-ALAS), is responsible for catalyzing a condensation reaction between succinyl-CoA and glycine to yield 5-aminolevulinic acid (ALA) (6). Using computational network studies, Bazzani et al. (3) identified *Pf* 5-ALAS as an essential enzyme with high selectivity during liver stage development, signifying its potential as a prophylactic antimalarial drug target. The essentiality of the pathway is likely due to the incorporation of heme into cytochromes, part of the *Pf* mitochondrial electron transport chain (7).

Even though heme-dependent electron transfer is vital for *Pf* asexual blood-stage replication, parasite heme biosynthesis appears not to be essential. This is likely due to parasites sequestering heme from host erythrocytes to fulfill the obligatory demand of heme for viable cytochromes b, c and c1 of the electron transport chain (8). This finding and reports that parasites lacking the *5-ALAS* gene can complete asexual blood stage replication show that *de novo* biosynthesis of heme is not essential during this life cycle stage (5). Inhibiting 5-ALAS, however, has been shown to strongly inhibit the progression of liver stage-to-blood stage transition and also prevent mosquito stage sporozoite maturation (9), and thus 5-ALAS has been suggested as a potential target for malaria prophylaxis and preventing malaria transmission (8).

Structure-based virtual screening (SBVS) presents a fast and cost-effective alternative approach to high throughput screening (HTS) in the drug development process (10). This technique can also improve the efficiency of lead compound selection by giving priority to compounds with a higher probability of success during the experimental testing phase (11). By combining pharmacophore modeling with molecular docking and *in silico* toxicity testing, computational approaches have proven to be potent in identifying potential drug compounds that may serve as leads in drug development (12–14). In this study, we aimed to identify important potential lead compounds which can serve as inhibitors of *Pf* 5-ALAS using pharmacophore modeling, virtual screening, qualitative structural assessment, *in silico* ADMET (Absorption, Distribution, Metabolism, Excretion and Toxicity) evaluation and Molecular Dynamics (MD) simulation.

## 2. Materials and methods

### 2.1. *Plasmodium falciparum* 5-ALAS structure prediction

The homology modeled 3D structure of *Pf* 5-ALAS was built via SWISS-MODEL using the structure of ALAS from *Saccharomyces cerevisiae* non-covalently bound to PLP cofactor (PDB ID: 5TXR), due to the absence of its experimental structure at the time of the research (15, 16). *Ab initio* models were also retrieved from AlphaFold (ID: AF-Q8I4 × 1-F1) and Robetta (17). Robetta is an automated online platform for both protein structure and function predictions based on the amino acid sequence of the protein. The amino acid sequence was retrieved from the Protein Database on the National Center for Biotechnology Information (NCBI) based with accession ID: XP_001350846.

### 2.2. Assessment of modeled 5-ALAS structures

Preliminary structure assessments of the predicted models of the protein were carried out using SWISS-MODEL (MolProbity and Clash scores) (18, 19), then ERRAT and VERIFY from the UCLA-DOE LAB – SAVES v6. The lower values of the MolProbity and clash score with higher percentiles in both cases indicate a better model. For the ERRAT score, an average overall quality factor of approximately 91% is a good model, while for VERIFY, at least 80% of the amino acids should score = 0.2 in the 3D/1D profile. The best model from the five models generated from Robetta was identified, while a further assessment was carried out on the best model and the AlphaFold model using MolProbity, http://molprobity.biochem.duke.edu/index.php. MolProbity is a web server employed in the validation of the quality of modeled protein and nucleic acid structures. The MolProbity score is a combined single indicator of model quality based on the clash, rotamer and Ramachandran parameters.

### 2.3. Alignment of the modeled *Pf* 5-ALAS structures

The alignment of the modeled 3D structures of *Pf* 5-ALAS from SWISS-MODEL, Robetta and AlphaFold was carried out using PyMOL. This was performed to measure the root mean square deviation (RMSD) between the three models using the best model from Robetta (due to the structure assessment) as the reference protein. RMSD indicates how close or similar the protein structure are. Normally, more similar proteins usually have small values of RMSD. RMSD is usually reported in Angstrom (Å).

### 2.4. Active site prediction of the 5-ALAS modeled structure

The active sites of the modeled 5-ALAS structure with the best structure assessment were predicted using Computed Atlas of Surface Topography of proteins (CASTp) 3.0 (20, 21) and the Prankweb server (22, 23). The modeled protein structure was submitted on the webservers. The necessary amino acids for binding interactions predicted by the two servers were compared to determine the similarity between the two predicted active sites.

### 2.5. Pharmacophore-based screening and ligand-library generation

A pharmacophore is a chemical framework that carries the structural features of a known active compound. A pharmacophore model is developed based on the structural features of an active compound and then employed in filtering, evaluating and screening databases of molecules (24). A ligand-based pharmacophore model based on the modeled structure of *Pf* 5-ALAS was developed using the Pharmit server^1^ (25, 26). The Pharmit server offers a set-up for the virtual screening of databases utilizing suitable pharmacophore features. The modeled *Pf* 5-ALAS was inputted as the receptor while the pharmacophore features were set based on the interaction of pyridoxal 5′-phosphate, a reported cofactor of *Pf* 5-ALAS (PubChem ID: 1051) in the binding site of the protein.

Four key properties of ligands in terms of hydrogen bond acceptors, hydrogen bond donors, hydrophobicity, and aromaticity were chosen to construct an effective pharmacophore query for virtual screening. The hit screening parameters were also set based on Lipinski’s rule of five (27): molecular weight ≤ 500, hydrogen bond acceptors ≤ 10, hydrogen bond donors ≤ 5, a logP (octanol-water partition coefficient) value ≤ 5 and Veber’s filter: rotatable bonds ≤ 10, Aromatics ≤ 2 and total polar surface area (TPSA) ≤ 140 Å. The following databases were explored for the pharmacophore-based virtual screening: CHEMBL, ChemDiv, ChemSpace, MCULE, MCULE-ULTIMATE, MolPort, NCI Open Chemical Repository, LabNetwork and ZINC databases.

### 2.6. Ligand preparation

The 2,755 compounds obtained from the pharmacophore-based screening were downloaded in their.sdf format and converted to their corresponding 3D structures using the OpenBabel (28) panel of PyRx (29) software. 2,621 compounds were successfully converted to the AutoDock docking format (.pdbqt) due to the inability to set up a force field for the remaining 134 compounds. Pyridoxal 5′-phosphate was also added to the ligand library of 2,621 compounds for the docking simulation.

### 2.7. Active site determination

The 5-ALAS active site was predicted using CastP and PrankWeb online servers. The active site residues generated from the prediction were used to construct a grid box around the active site using 100, 100, and 100 as the number of points in x, y, and z directions. A grid point spacing of 0.375 Å was employed, with the center grid box set at 31.369, 26.246, and 6.703 Å of the modeled *Pf* 5-ALAS structure.

### 2.8. Virtual screening and post-screening analyses

The molecular docking analysis was performed on Autodock vina installed on the Covenant University Bioinformatics Research (CUBRe) high-performance computer (HPC). The post docking analysis was carried out using the Discovery Studio 2021 Client (30). The structural representations of the best hits in relation to the binding affinities were also examined to build the SAR (Structure-Activity Relationship) studies. SAR refers to approaches built to find relationships between chemical structures of studied compounds and biological activity (or target property). The extensive qualitative structural assessment was carried out on the four best hits to examine the heterocyclic templates and functional groups responsible for the different binding interactions observed in the post-screening analysis.

### 2.9. *In silico* ADMET prediction

The increased drug attrition rate has been attributed to toxicity and poor pharmacokinetics (31). ADMET prediction is employed in estimating the pharmacokinetic properties and toxicity risk (32). This also predicts if proposed lead compounds stand the chance of being orally active drugs. The OSIRIS Property Explorer tool was employed in this study for the prediction of ADMET of the best hits. The following pharmacokinetic properties were examined: molecular weight, solubility (log S), hydrophilicity (log P), topological polar surface area (TPSA), drug-likeness, and drug score. Furthermore, the toxicity risks of the compounds in the form of tumorigenic, mutagenic, irritating, and reproductive risks were also examined.

### 2.10. Molecular dynamics simulation

The MD simulation was carried out by using NAMD (Nanoscale Molecular Dynamics) (33) and VMD (Visual Molecular Dynamics) (34). This helped to examine the stability of the best confirmation of **CSMS00081585868** from the docking studies in the binding site of the modeled *Pf* 5-ALAS. Energy minimization of 1,000 steps and production run of 5,00,000 steps (1 ns) were employed in the simulation. The topology of the target was generated using VMD while that of ligand built using Charmm36 forcefield of the Charmm-GUI webserver. The topologies (pdb and psf) of the *Pf* 5-ALAS and **CSMS00081585868** were merged, and the complex was solvated using VMD. The simulation was performed at constant pressure of 1 atm and temperature of 310 K using Periodic Boundary conditions. All the necessary parameters for the simulation were defined in a script and executed using NAMD. The RMSD (root mean square deviation), RMSF (root mean square fluctuation) and PCA (principal component analysis) of the simulation results were carried out using Bio3D on the Galaxy Europe platform (35) while the hydrogen bond (h-bond) analysis was carried out using VMD software.

## 3. Results

### 3.1. Assessment of modeled 5-ALAS structure

Structure assessment of the *Pf* 5-ALAS models obtained from SWISS-MODEL, Robetta and AlphaFold revealed that the *Robetta* modeled structure was the best based on the different assessment scores. Robetta generated five models of the protein with a confidence score of 0.67. Modeled structure (Robetta_3) was selected as the best model based on the parameters obtained from the structure assessments. Robetta_3 had a MolProbity score of 1.47 (96th percentile), clash score of 3.93 (96th percentile), ERRAT score of 94.53%, and VERIFY score of 82.70% (Table 1). However, further structure assessment of the modeled Robetta_3 in comparison with the AlphaFold model showed that modeled Robetta_3 is better (Table 2). Robetta_3 had 95.86% of the amino acids in the Ramachandran favored region and 0.64% as Ramachandran outliers, while the AlphaFold model had 93.23% of the amino acids in the Ramachandran favored region and 5.25% as Ramachandran outliers.

**TABLE 1:** Comparison of the structure assessments of the modeled *Pf* 5-ALAS 3D structures based on SWISS-MODEL, ERRAT and VERIFY.

	SWISS-MODEL ASSESSMENT		UCLA-DOE LAB – SAVES v6	
**Models**	**MolProbity score**	**Clash score**	**ERRAT (%)**	**VERIFY (%)**
SWISS-MODEL	1.83 (84th percentile)	4.34 (96th percentile)	85.31	70.37 (Fail)
AlphaFold	1.86 (82nd percentile)	1.72 (99th percentile)	90.82	66.03 (Fail)
Robetta_1	1.67 (90th percentile)	6.23 (90th percentile)	91.77	79.84 (Fail)
Robetta_2	1.58 (93rd percentile)	3.93 (96th percentile)	95.49	77.94 (Fail)
Robetta_3	1.47 (96th percentile)	3.93 (96th percentile)	94.53	82.70 (Pass)
Robetta_4	1.58 (93rd percentile)	3.93 (96th percentile)	92.41	84.92 (Pass)
Robetta_5	1.74 (88th percentile)	4.98 (94th percentile)	91.07	80.00 (Pass)

**TABLE 2:** Further structure assessment evaluation of the AlphaFold generated structure and the best model from Robetta webserver using MolProbity.

	Parameters	AlphaFold (AF-Q8I4 × 1-F1)		Robetta_3		
All-atom contacts	Clashscore, all atoms:	1.72 (99th percentile)		3.93 (96th percentile)		
Protein geometry	Poor rotamers	17	2.88%	0	0.00%	Goal: 0.3%
	Favored rotamers	551	93.23%	0	0.00%	Goal: >98%
	Ramachandran outliers	33	5.25%	4	0.64%	Goal: <0.05%
	Ramachandran favored	555	88.38%	602	95.86%	Goal: >98%
	Rama distribution *Z*-score	–2.07 ± 0.30		–0.39 ± 0.33		Goal: abs (*Z* score) < 2
	MolProbity score	1.86 (82nd percentile)		1.47 (96th percentile)		
	Cβ deviations > 0.25Å	9	1.49%	1	0.17%	Goal: 0
	Bad bonds:	0/270	0.00%	9/5270	0.17%	Goal: 0%
	Bad angles:	62/7098	0.87%	24/7098	0.34%	Goal: < 0.1%
Peptide omegas	Cis prolines:	1/11	9.09%	1/11	9.09%	Expected: ≤ 1 per chain, or ≤ 5%
	Cis non-prolines:	2/618	0.32%	5/618	0.81%	
	Twisted peptides:	34/629	5.41%	**–**	**–**	Goal: 0
Low-resolution criteria	CaBLAM outliers	30	4.8%	21	3.4%	Goal: < 1.0%
	CA geometry outliers	24	3.83%	10	1.60%	Goal: < 0.5%
Additional validations	Chiral volume outliers	0/790		0/790		

### 3.2. Alignment of the modeled *Pf* 5-ALAS structures

**FIGURE 1:**
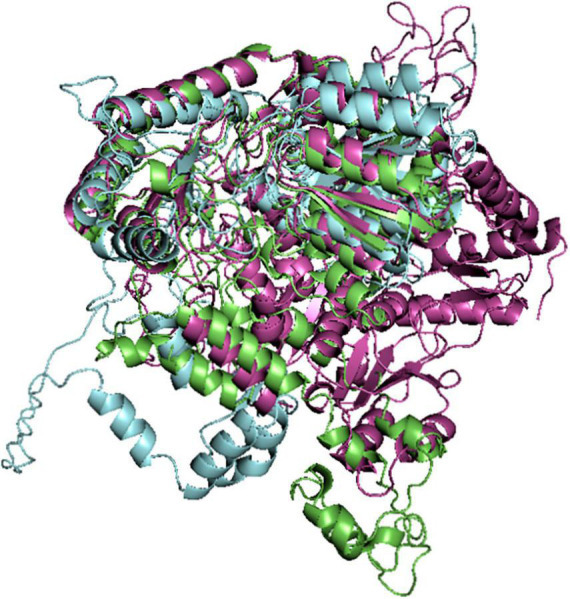
Alignment of the modeled *Pf* 5-ALAS Structures from Robetta (green colored), SWISS-MODEL (purple colored) and AlphaFold (blue colored).

The RMSD of the AlphaFold modeled structure was obtained to be 12.894, having 3,978 atoms different from that of reference model. Furthermore, SWISS-MODEL structure had RMSD value of 2.053 and 3,200 atoms different from the reference model. This suggested that the homology modeled structure from SWISS-MODEL was more similar to that of the reference Robetta model than that of the AlphaFold model (Figure 1).

### 3.3. Pharmacophore-based screening and ligand-library generation

The pharmacophore features of pyridoxal 5′-phosphate gave rise to the generation of the parameters used in the pharmacophore-based modeling on the Pharmit server. The x, y, and z coordinates of the different features (Hydrogen donor, Hydrogen acceptor, Aromatics and Hydrophobic) are shown in Table 3. The pharmacophore-based screening of the different databases (CHEMBL30, ChemDiv, ChemSpace, MCULE, MCULE-ULTIMATE, MolPort, NCI Open Chemical Repository, LabNetwork and ZINC) gave rise to 2,755 hits (Table 4). The total number of hits obtained from the databases are in the order: ChemSpace > ZINC > MCULE > MolPort > CHEMBL30 > ChemDiv > LabNetwork > NCI Open Chemical Repository > MCULE-ULTIMATE.

**TABLE 3:** Parameters employed in setting the pharmacophore features on Pharmit.

Pharmacophore features	*x*	*y*	*z*	Radius
Hydrogen donor	3.26	-1.39	-0.5	0.5
Hydrogen acceptor	2.08	2.0	0.15	0.5
Hydrogen acceptor	-1.97	0.33	-0.43	0.5
Hydrogen acceptor	3.26	-1.39	-0.5	0.5
Hydrogen acceptor	1.25	-2.96	0.36	0.5
Aromatic	1.57	0.69	0.14	1.1
Hydrophobic	1.57	0.69	0.14	1.0

**TABLE 4:** Total number of hits generated from the pharmacophore-based screening.

S.No	Databases	Hits
1	CHEMBL30	298
2	ChemDiv	66
3	ChemSpace	662
4	MCULE	524
5	MCULE-ULTIMATE	1
6	MolPort	461
7	NCI Open Chemical Repository	32
8	LabNetwork	56
9	ZINC	655
	**Total:**	**2,755**

### 3.4. Virtual screening analyses

A total of 2,621 compounds, including pyridoxal 5′-phosphate, were docked into the predicted active site of *Pf* 5-ALAS, and the best hits were obtained (Table 5). The binding affinities of the top ten hits from the virtual screening were between -9.9 and -9.1 kcal/mol, while the predicted inhibition constant (Ki) was within the ranges of 52.10 and 202.01 nM. All the top ten hits had better binding affinities than the cofactor pyridoxal 5′-phosphate, with a docking score of -6.4 kcal/mol. However, compound **CSMS00081585868**, obtained from the Chemspace database, had the best binding affinity with a docking score of -9.9 kcal/mol and Ki of 52.10 nM. This means that this compound has the highest probability of causing inhibition of *Pf* 5-ALAS, thereby limiting the activity of the parasite at the liver stage.

**TABLE 5:** The structures, binding affinities and predicted Ki of the ten best hits from the virtual screening alongside the naturally occurring cofactor pyridoxal 5′-phosphate.

S/N	Compound codes	Structures	Binding affinities (kcal/mol)	Predicted Ki (nM)
1	**CSMS00081585868** (Chemspace ID)	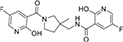	-9.9	52.10
2	**MolPort-047-716-699** (MolPort ID)	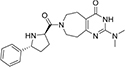	-9.6	86.61
3	**CSCS00058497692** (Chemspace ID)	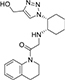	-9.5	102.59
4	**MolPort-047-733-181** (MolPort ID)	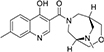	-9.4	121.53
5	**ZINC5276997** (ZINC ID)	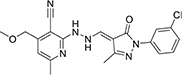	-9.2	170.54
6	**CSMS00083851644** (Chemspace ID)	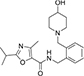	-9.2	170.54
7	**MolPort-039-017-789** (MolPort ID)	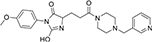	-9.2	170.54
8	**ZINC65533569** (ZINC ID)	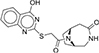	-9.2	170.54
9	**MolPort-046-194-001** (MolPort ID)	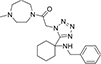	-9.1	202.01
10	**MolPort-045-962-768** (MolPort ID)	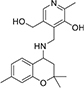	-9.1	202.01
11	**pyridoxal 5**’**-phosphate**	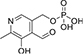	-6.4	19,574.84

Ki- inhibition constant.

### 3.5. Post-screening analyses

The interactions of the atoms of the best hits with the amino acid residues in the active site of *Pf* 5-ALAS were analyzed using Discovery Studio 2021 client. The interactions observed from the post-screening analyses include conventional hydrogen bond, carbon-hydrogen bond, pi-cation, pi-anion, pi-sigma, pi-pi stacked, alkyl and pi-alkyl. These interactions and bond lengths are presented in Table 6. All these interactions contribute significantly to the binding affinities and docking score obtained for each of the best hits. The presence of intermolecular hydrogen bonds formed between the ligand and the amino acids in the active sites contributes significantly to the strength of the complex formed, which invariably impacts the docking result positively (36). Hence, the availability of hydrogen bond acceptors (HBA) and hydrogen bond donors (HBD) in the structure of the ligand is important. According to Lipinski’s rule of five (37), a drug candidate should possess HBA ≤ 10 and HBD ≤ 5. **CSMS00081585868**, the best hit from the screening, has eight HBA and three HBD, which were responsible for seven hydrogen bonds between the atoms of the compound and the amino acid residues in the *Pf* 5-ALAS active site. The hydrogen bonds were formed at SER 43, ASP 61, LYS 63, ASN 331, TYR 328, ASP 430, and HIS 433, and these interactions contributed to the high binding affinity observed for the compound. Likewise, the atoms of **MolPort-047-716-699** formed different interactions with the amino acid residues in the *Pf* 5-ALAS active site, which include carbon-hydrogen bond at SER 234, amide-pi stacked/pi-pi stacked interaction at ASN 194 and TYR 202, alkyl/pi-alkyl interaction at PRO 212, LYS196 and TYR 202. Similarly, **CSCS00058497692,** with five HBA and two HBD, formed three hydrogen bonds with LEU 462, GLY 470 and SER 463 in the *Pf* 5-ALAS active site. More interactions formed include pi-donor hydrogen bond at SER 326, pi-cation/pi-anion interaction with LYS 63 and ASP 44. Furthermore, pi-pi stacked interaction was also observed at TYR 328, while alkyl/pi-Alky interaction was observed at LEU 469. Furthermore, **MolPort-047-733-181** formed three hydrogen bonds with the *Pf* 5-ALAS active site at SER 40, SER 43 and ASN 62. Pi-cation/pi-anion interactions were also observed at LYS 65 and ASP 44 while pi-pi stacked interaction was observed at TYR 328 and alkyl/pi-alkyl interactions at VAL 48, ILE 68, PRO 47, TYR 328.

**TABLE 6:** The interactions and bond lengths of the ten best hits in the active site alongside the naturally occurring cofactor pyridoxal 5′-phosphate.

S/N	Compound codes	Hydrogen bond acceptors	Hydrogen bond donors	Interactions and bond lengths
1	**CSMS00081585868** (Chemspace ID)	8	3	**Conventional Hydrogen Bond:** ASP 430 (2.20 Å), ASN 331 (2.91 Å), LYS 63 (5.86 Å), SER 43 (2.39 Å), TYR 328 (3.09 Å), HIS 433 (2.59 Å) **Halogen:** ASP 61 (2.20 Å)
2	**MolPort-047-716-699** (MolPort ID)	4	2	**Carbon Hydrogen Bond:** SER 234 (3.08 Å) **Amide-Pi stacked/Pi-Pi Stacked:** ASN 194 (4.17 Å), TYR 202 (5.19 Å) **Alkyl/Pi-Alkyl:** PRO 212 (5.04 Å), LYS 196 (5.01 Å, 5.31 Å), TYR 202 (4.97 Å) **Van der Waals:** LYS 195
3	**CSCS00058497692** (Chemspace ID)	5	2	**Conventional Hydrogen Bond:** LEU 462 (2.93 Å), GLY 470 (1.86 Å), SER 463 (2.22 Å) **Pi-Donor Hydrogen Bond:** SER 326 (2.74 Å) **Pi-Cation/Pi-Anion:** LYS 63 (4.52 Å), ASP 44 (3.64 Å) **Pi-Pi Stacked:** TYR 328 (3.70 Å) **Alkyl/Pi-Alkyl:** LEU 469 (4.89 Å, 5.07 Å)
4	**MolPort-047-733-181** (MolPort ID)	5	1	**Conventional Hydrogen Bond:** ASN 62 (2.31 Å), SER 43 (2.16 Å) **Carbon Hydrogen Bond:** SER 40 (4.12 Å) **Pi-Cation/Pi-Anion:** LYS 65 (5.13 Å), ASP 44 (5.08 Å) **Pi-Pi Stacked:** TYR 328 (3.70 Å) **Alkyl/Pi-Alkyl:** VAL 48 (4.99 Å), ILE 68 (4.47 Å), PRO 47 (4.11 Å), TYR 328 (5.13 Å)
5	**ZINC5276997** (ZINC ID)	5	2	**Conventional Hydrogen Bond:** ASN 62 (4.31 Å), SER 43 (3.16 Å, 3.76 Å) **Carbon Hydrogen Bond:** VAL 41 (4.12 Å) **Pi-Cation/Pi-Anion:** LYS 63 (5.89 Å), ASP 44 (4.77 Å, 5.83 Å) **Pi-Sigm** TYR 328 (4.42 Å) **Pi-Pi Stacked:** TYR 328 (4.90 Å) **Alkyl/Pi-Alkyl:** LEU 27 (5.98 Å), LEU 469 (5.08 Å), CYS 213 (5.71 Å)
6	**CSMS00083851644** (Chemspace ID)	5	2	**Conventional Hydrogen Bond:** LYS 63 (5.31 Å), SER 43 (4.82 Å), SER 463 (3.65 Å) **Pi-Cation/Pi-Anion:** LYS 464 (6.91 Å), ASP 44 (5.05 Å) **Pi-Pi Stacked:** TYR 328 (4.41 Å)
7	**MolPort-039-017-789** (MolPort ID)	7	1	**Conventional Hydrogen Bond:** SER 463 (3.81 Å) **Carbon Hydrogen Bond:** SER 40 (4.63 Å), GLU 401 (5.35 Å) **Alkyl/Pi-Alkyl:** VAL 432 (5.92 Å), LEU 469 (4.56 Å), HIS 433 (4.95 Å)
8	**ZINC65533569** (ZINC ID)	5	2	**Conventional Hydrogen Bond:** SER 43 (3.37 Å), LYS 464 (5.83 Å, 6.10 Å) **Pi-Cation/Pi-Anion:** LYS 63 (5.85 Å), ASP 44 (6.03 Å) **Alkyl/Pi-Alkyl:** LEU 469 (4.12 Å) **Pi-Pi Stacked:** TYR 328 (5.23 Å)
9	**MolPort-046-194-001** (MolPort ID)	6	1	**Conventional Hydrogen Bond:** ARG 174 (6.15 Å), HIS 353 (5.72 Å) **Pi-Cation/Pi-Anion:** LYS 65 (6.90 Å), ASP 44 (5.28 Å) **Pi-Pi Stacked:** TYR 328 (4.74 Å)
10	**MolPort-045-962-768** (MolPort ID)	5	3	**Conventional Hydrogen Bond:** ASN 62 (4.04 Å), LYS 464 (5.85 Å), LYS 462 (5.73 Å), GLY 470 (2.99 Å) **Pi-Cation/Pi-Anion:** LYS 63 (6.49 Å), ASP 44 (5.08 Å) **Pi-Pi Stacked:** TYR 328 (4.27 Å) **Alkyl/Pi-Alkyl:** LYS 63 (4.88 Å), HIS 353 (6.06 Å)
11	**Pyridoxal 5**’**-phosphate**	7	3	**Conventional Hydrogen Bond:** SER 43 (4.14 Å), LYS 464 (6.07 Å), ASN 62 (4.05 Å) **Pi-Alkyl:** TYR 328 (4.81 Å)

### 3.6. Qualitative structural assessment of the four best hits in the docking model

The structural representations of the four best hits from the virtual screening were further examined to understand their structure-activity relationship (SAR). This was carried out to understand the significant heterocyclic templates and functional groups present on the compounds that contributed to the interactions formed between the atoms of the compounds and the active site of the *Pf* 5-ALAS. With the understanding of the SAR of the best hits, new scaffolds can be designed that can stand a chance as new potential antimalarial prophylaxis drugs against *Pf* 5-ALAS. Also, functional group interconversion can be employed with careful hit-to-lead optimization. The qualitative structural assessments of the best five hits from the virtual screening are discussed below:

#### 3.6.1. CSMS00081585868

**FIGURE 2:**
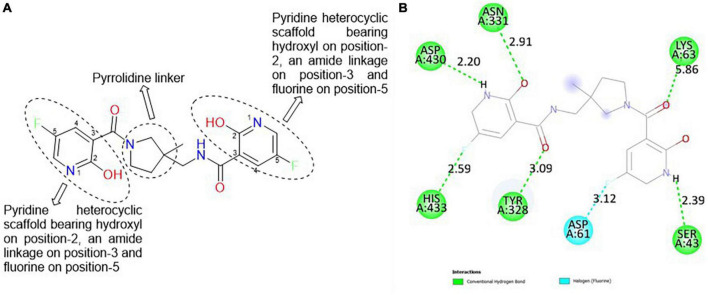
**(A)** Qualitative structural assessment of CSMS00081585868. **(B)** Post docking two-dimensional visualization of CSMS00081585868 from the virtual screening in the binding pockets of *Pf* 5-ALAS.

The best hit, **CSMS00081585868**, comprises two pyrazine rings linked together by a pyrrolidine template (Figure 2A). Pyrrolidine (also known as tetrahydropyrrole) is a nitrogen-containing five-membered heterocyclic compound, while pyridine is a six-membered ring nitrogen-containing heterocyclic compound. The two pyridine rings possess hydroxyl (OH) on position-2, an amide linkage on position-3 and fluorine (F) on position-5. The O of the OH on one of the pyrazine rings formed a hydrogen bond with ASN 331 at a bond length of 2.91 Å, while the H of the NH on both pyrazine rings formed hydrogen bonds with ASP 430 and SER 43 at bond lengths of 2.20 and 2.39 Å, respectively. The presence of the fluorine atoms on the pyrazine rings also contributed significantly to the binding affinities forming strong hydrogen bonds with SER 43 and HIS 433 at bond lengths of 2.39 and 2.59 Å. The two O of the amide functional groups on the compound formed hydrogen bonds with LYS 63 and TYR 328 at bond lengths of 5.86 and 3.09 Å (Figure 2B).

#### 3.6.2. MolPort-047-716-699

**FIGURE 3:**
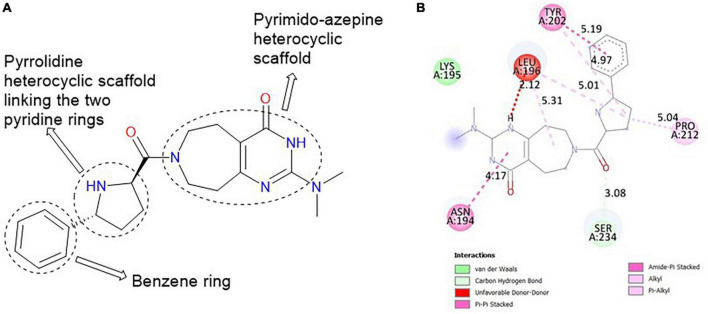
**(A)** Qualitative structural assessment of MolPort-047-716-699. **(B)** Post docking two-dimensional visualization of MolPort-047-716-699 from the virtual screening in the binding pockets of *Pf* 5-ALAS.

The major heterocyclic templates in the structure of **MolPort-047-716-699** are pyrimido-azepine and pyrrolidine, linked by a ketone (C=O) functional group (Figure 3A). The pyrimido-azepine is a fusion of pyrimidine (a six-membered ring heterocyclic compound with nitrogen on position-1 and 3) and hexahydro-1H-azepine (a saturated seven-membered ring nitrogen-containing heterocyclic compound. The O of the C=O linkage formed a carbon-hydrogen bond with SER 234 at a bond length of 3.08 Å. A van der waals interaction was also observed between the compound and LYS 195 (Figure 3B).

#### 3.6.3. CSCS00058497692

**FIGURE 4:**
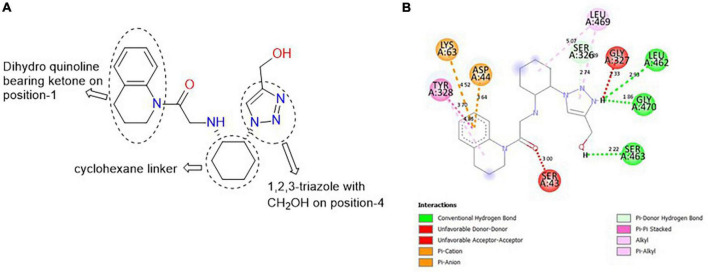
**(A)** Qualitative structural assessment of CSCS00058497692. **(B)** Post docking two-dimensional visualization of CSCS00058497692 from the virtual screening in the binding pockets of *Pf* 5-ALAS.

**CSCS00058497692**, comprises two heterocyclic rings: dihydroquinoline (fusion of benzene and piperidine rings) and 1,2,3-triazole (five-membered ring with nitrogen at position-1, 2 and 3). These rings are linked together by a cyclohexane template (Figure 4A). The H of the CH_2_OH on position-4 of the triazole is responsible for the hydrogen bond formed with SER 463 at a bond length of 2.22 Å. The H of the N on position-3 of the triazole is responsible for the hydrogen bonds formed with LEU 462 and GLY 470 at 2.93 and 1.86 Å, respectively (Figure 4B).

#### 3.6.4. MolPort-047-733-181

**FIGURE 5:**
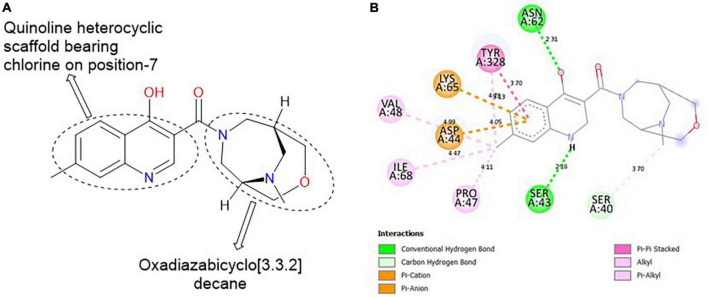
**(A)** Qualitative structural assessment of MolPort-047-733-181. **(B)** Post docking two-dimensional visualization of MolPort-047-733-181 from the virtual screening in the binding pockets of *Pf* 5-ALAS.

**MolPort-047-733-181**, comprises two heterocyclic rings: quinoline (fusion of benzene and pyridine rings) and oxadiazabicyclo[3.3.2]decane. These rings are linked together by a carbonyl group (C=O; Figure 5A). The H of the NH of quinoline formed a hydrogen bond with SER 43 at a bond length of 2.16 Å, while the O of the OH on position-4 of the quinolone formed a hydrogen bond with ASN 62A at 2.31 Å. The O of the oxadiazabicyclo[3.3.2]decane is responsible for the carbon-hydrogen bond formed with SER 40 at a bond length of 3.70 Å. Furthermore, the methyl group on position-7 of the quinoline template formed alkyl/pi-alkyl interactions with VAL 48, ILE 68, and TYR 328 at bond lengths of 4.99, 4.47, and 5.13 Å, respectively (Figure 5B).

### 3.7. *In silico* ADMET prediction

The pharmacokinetic properties and toxicity risks of all the best hits, as predicted by OSIRIS Property Explorer, are presented in Table 7. Molecular weights (MW) of compounds determine their ease of distribution within cells. Compounds with lesser MW tend to be more easily distributed than compounds with higher MW, hence the benchmark of 500 g/mol. All the best hits had MWs within the acceptable range (between 341.0 and 437.5 g/mol). The logarithm of the partition coefficient between n-octanol and water is used to determine the clog P, which is a measure of a compound’s hydrophilicity or lipophilicity. A clog P of more than 5.0 indicates low hydrophilicity or poor absorption, but clogP values less than 5.0 are acceptable. All the best hits possessed clog P values less than 5.0, which suggests good absorption potentials for all the compounds. The topological polar surface area (TPSA) of a molecule is strongly related to its hydrogen bonding and is a good predictor of bioavailability (38). A score of less than 160 Å^2^ is considered acceptable for TPSA, indicating that the molecule will have good oral bioavailability (39). All the best hits had acceptable TPSA scores.

**TABLE 7:** Pharmacokinetic properties and toxicity risks of the 10 best hits as estimated using the OSIRIS property explorer tool.

Compound codes		Pharmacokinetic properties					Toxicity risks			
	**MW**	**cLogP**	**TPSA (Å^2^)**	**Solubility Prediction** ** (log S)**	**Drug likeness**	**Drug score**	**Mutagenic**	**Tumorigenic**	**Irritant**	**Reproductive** **effective**
CSMS00081585868	392.0	1.50	115.60	-2.71	3.67	0.83	Passed	Passed	Passed	Passed
MolPort-047-716-699	381.0	1.20	77.04	-2.46	7.66	0.86	Passed	Passed	Passed	Passed
CSCS00058497692	369.0	1.29	83.28	-2.56	-3.59	0.36	Passed	Passed	Mild risk	Passed
MolPort-047-733-181	341.0	1.58	65.90	-1.86	2.62	0.52	Passed	Fail	Passed	Passed
ZINC5276997	410.0	3.20	102.60	-4.61	0.47	0.52	Passed	Passed	Passed	Passed
CSMS00083851644	371.0	2.52	78.60	-3.01	5.01	0.82	Passed	Passed	Passed	Passed
MolPort-039-017-789	437.0	1.14	98.57	-2.44	7.11	0.80	Passed	Passed	Passed	Passed
ZINC65533569	358.0	1.54	120.70	-3.32	2.97	0.82	Passed	Passed	Passed	Passed
MolPort-046-194-001	411.0	1.46	79.18	-1.76	1.59	0.77	Passed	Passed	Passed	Passed
MolPort-045-962-768	342.0	2.29	74.61	-3.01	-3.32	0.44	Passed	Passed	Passed	Passed

Solubility is also a significant consideration in pharmacokinetics as it impacts both absorption and distribution. It is measured as a logarithm of the solubility measured in mol/dm^3^. All the compounds possess estimated log S values greater than -4 except for **ZINC5276997**, and this corresponds to the score of more than 80% of marketed drugs. The drug-likeness of the compounds is predicted on a positive or negative basis. A positive number indicates that the molecule has a high percentage of fragments that are often seen in marketed drugs. All the ten best hits had positive values except for **CSCS00058497692** and **MolPort-045-962-768** with -3.59 and -3.32, respectively. The drug score parameter combines drug-likeness, cLogP, logS, molecular weight, and toxicity risk into one easy-to-understand number that may be used to assess a compound’s overall potential to become a drug. The higher the drug score value, the higher the compound’s chance of being considered a drug candidate (40). Compound **MolPort-047-716-699** had the highest drug score with a value of 0.86. Furthermore, the toxicity properties evaluated were color-coded green, yellow or red. The properties displayed in red suggest a severe danger of unwanted consequences, yellow suggests mild toxicity, whereas the properties given in green imply drug conformity, compatibility, and safety *in vivo*. The toxicity results indicated that all the compounds showed no mutagenic or irritant risk. However, **MolPort-047-733-181** was predicted to have high tumorigenic tendencies, while **CSCS00058497692** with a medium irritant risk.

### 3.8. Molecular dynamics simulation

#### 3.8.1. Root mean square deviation ligand and root mean square fluctuation

**FIGURE 6:**
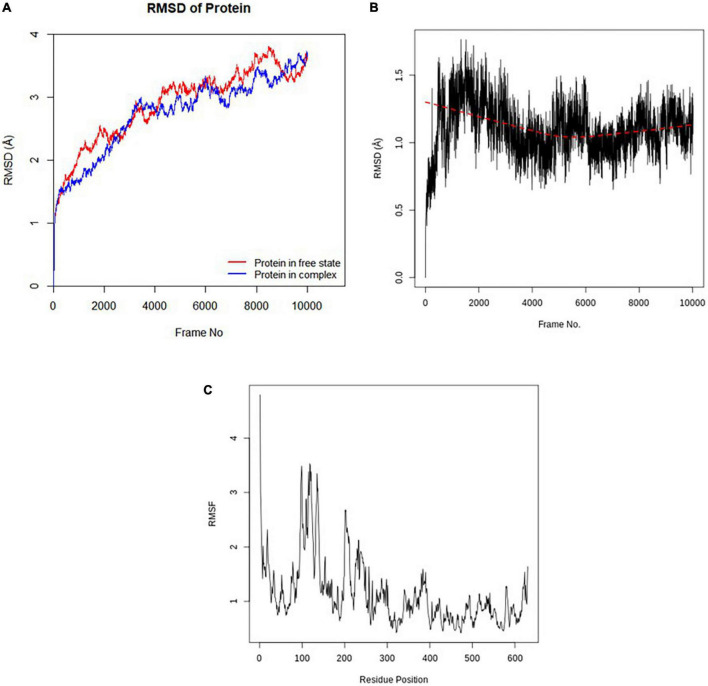
**(A)** RMSD plot of the c-alpha protein backbone (without ligand and in complex with ligand). **(B)** RMSD plot of CSMS00081585868 in the active site of *Pf* 5-ALAS. **(C)** RMSF plot of the c-alpha protein backbone.

The stabilities of the c-alpha of the *Pf* 5-ALAS backbone without the ligand and in the protein-ligand complex as well as that of the **CSMS00081585868** ligand in the complex were depicted in the RMSD plots obtained from the molecular dynamics (MD) simulation studies (Figures 6A–C). The RMSD of the two states of the c-alpha protein backbone fluctuated between 1.5 and 4.0 Å, with an average RMSD less that 3 Å. This confirmed that the conformation of the protein remained relatively stable throughout the MD simulation even with the binding of the ligand in its active site (Figure 6A). The RMSD of **CSMS00081585868** fluctuated around 1.2 Å over the 10,020 frames after an initial increase from 0.0 Å, suggesting that the compound do not significantly change its orientation during the simulation (Figure 6B). The RMSF plot of the c-alpha protein backbone is shown in Figure 6C. A total of 630 residue positions were observed, while the highest RMSF values after simulation were observed at positions 118 and 119.

#### 3.8.2. Principal component analysis

**FIGURE 7:**
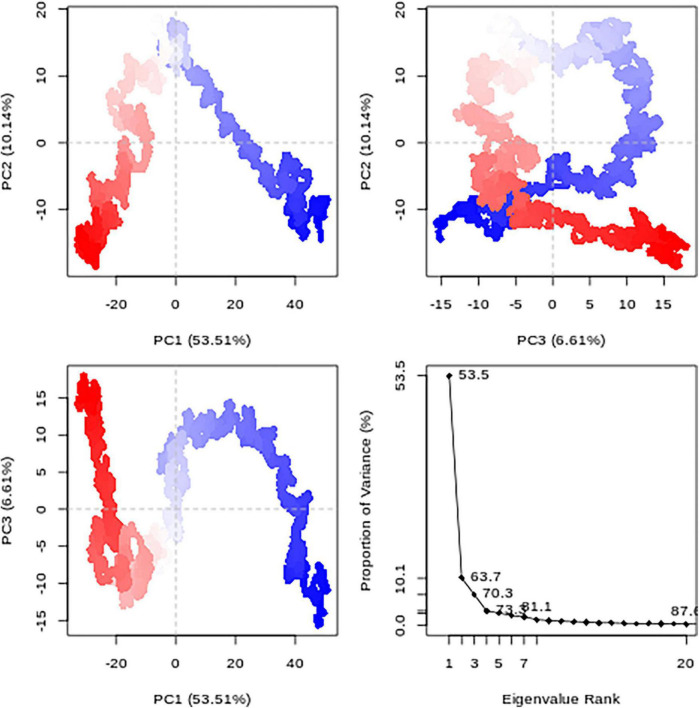
The principal component analysis (PCA) results for each data point (colored from blue to red according to time series) and the Eigenvalue rank plot.

The correlation of the motion of *Pf* 5-ALAS protein atoms are converted to a group of principal components which are uncorrelated, and this is captured in the form of PCA plots (Figure 7). The first PC (PC1) accounted for 53.5% of the cumulative variance, while PC2 and PC3 were responsible for 10.14 and 6.61%, respectively, as seen from the eigenvalue rank plot.

#### 3.8.3. Hydrogen bond analysis

**FIGURE 8:**
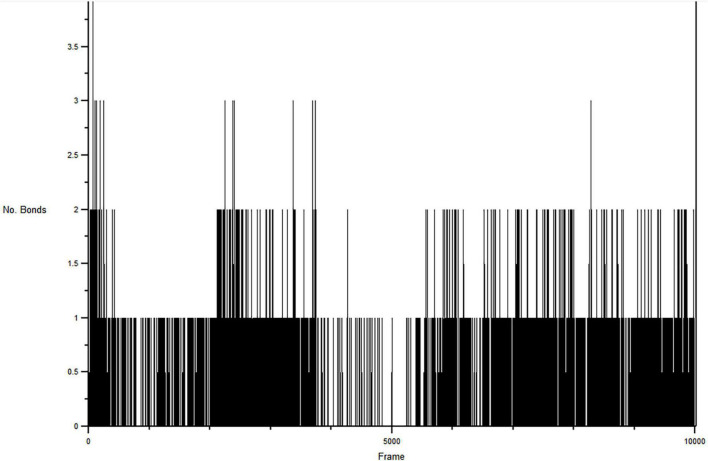
Hydrogen bonds formed between CSMS00081585868 in the active site of *Pf* 5-ALAS during the production run.

H-bond formations are important in the stabilities of the complexes during MD simulation. The number of H-bonds observed between the atoms of **CSMS00081585868** and the amino acid residues in the protein binding pockets during the simulation are shown in Figure 8. A total of 9 h-bonds was found during the MD simulation while the highest occupancy rate (19.93%) was observed for the interaction between **CSMS00081585868** as the donor and ASP 430 as the acceptor. This corresponded with the observed h-hond interaction between the hydrogen of the NH on pyridine in **CSMS00081585868** structural template and ASP 430 of *Pf* 5-ALAS at bond length of 2.20 Å from the docking studies (as seen in Figure 2B).

## 4. Discussion

Most of the available chemotherapeutic interventions for the treatment of malaria are designed against the erythrocytic stage, while only a few works have been done regarding the liver stage (41). This has been attributed to little knowledge about the biology of the malaria parasite, especially the deadly *Plasmodium falciparum* (42, 43). In the quest to get possible drug targets essential at the liver stage, *Pf* 5-ALAS model was developed using the *ab initio modeling* approach (44). The predicted model generated using the Robetta server (Figure 1) gave a better structure assessment result than the AlphaFold model (Table 2). Previous studies have also reported that in some cases, the Robetta model provided a better structural confidence score than the AlphaFold model (45). The docking scores from the virtual screening of downloaded compounds from nine different databases against the *Pf* 5-ALAS model showed that all the ten best hits possessed better binding affinity than the reported co-ligand, pyridoxal 5′-phosphate (6). The post-screening analysis carried out also helped to identify the binding interactions and bond lengths between the atoms of the best hits and the amino acid residues in the protein’s active site. **CSMS00081585868** had the best ligand efficiency with a docking score of -9.9 kcal/mol and predicted Ki of 52.10 nM, engaging in seven hydrogen bonds with the target’s active site amino acid residues. The top ten hits had binding affinities in the order: **CSMS00081585868** > **MolPort-047-716-699** > **CSCS00058497692** > **MolPort-047-733-181** > **ZINC5276997** > **CSMS00083851644** > **MolPort-039-017-789** > **ZINC65533569** > **MolPort-046-194-001** > **MolPort-045-962-768** (Table 5). The docking scores can be attributed to the binding interactions observed between the atoms of the best hits and the binding pocket of the modeled target (24; Table 6).

The pharmacophore features of a compound go a long way to determining whether the compound will have good interactions with the target (46, 47). Heterocyclic compounds are important pharmacophores in drug design as over 90% of commercially-available drugs have them embedded in their structure, and they have a wide range of therapeutic applications (48). Hence, the four best hits from this study were examined to understand the structural components in their backbone responsible for the interactions observed (Figures 2–5). All the four best hits possessed heterocycles as major backbones in their structures, with the pyrrolidine template observed as a major linker in **CSMS00081585868** (Figure 2) and **MolPort-047-716-699** (Figure 3). The qualitative structural assessment of the best hit, **CSMS00081585868**, revealed its drug-like configuration, with two pyridine heterocyclic scaffolds bearing hydroxy and fluorine atoms linked by a pyrrolidine heterocyclic scaffold.

Drug attrition is one of the significant problems in medicinal research, as most proposed drug candidates at the discovery phase end up not being translated into marketable drugs (31). This has been attributed to poor pharmacokinetics and high toxic risk (49). Hence, *in silico* ADMET study was conducted on the best hits, and it was observed that all the compounds had good pharmacokinetic properties according to Lipinski’s rule of five (37). The drug scores of the ten best hits were in the order: **MolPort-047-716-699** > **CSMS00081585868** > **CSMS00083851644** = **ZINC65533 569** > **MolPort-046-194-001** > **MolPort-047-733-181 = ZINC 5276997 > CSMS00083851644** > **MolPort-045-962-768** > **CSCS00058497692**. For the toxicity risk, **MolPort-047-733-181** and **CSCS00058497692** had high tumorigenic tendencies and medium irritant risk, respectively (Table 7). However, careful hit-to-lead optimization can be carried out on these compounds to make them non-toxic, as toxicity is usually a result of the presence of an unwanted functional group or pharmacophore present in the structure of the compound (50).

The stability of the best hit, **CSMS00081585868**, in the active site of *Pf* 5-ALAS and that of the c-alpha backbone of the protein were determined using MD simulation via the RMSD and RMSF analyses (Figure 6), PCA (Figure 7) and hydrogen bond analysis (Figure 8). Calculation of the RMSDs of the ligand and c-alpha protein backbone are important to identify the ligand’s possible modes of binding (51). The RMSD plot the **CSMS00081585868** in the active site suggest that the ligand is stable with a steady fluctuation around 1.2 Å (52). The stabilities of both the ligand and protein during the MD simulation process showed that a stable complex was formed. A comparison of the c-alpha protein backbone without the ligand and in complex with the ligand showed that the RMSD observed from the complexed protein was due to the ligand binding. Inspection of the *Pf* 5-ALAS surface during simulation, using VMD, also showed that the major fluctuations especially those with RMSF above 1.0 Å were from the flexible loop regions (53). The variance proportion resulting from each principal component (PC) was also observed from the eigenvalue rank plot (Figure 7). The PCA study showed that the first three principal components accounted for 70.3% of the total variance while the hydrogen bond analyses (Figure 8) showed that the ligand maintained stable conformation in the active site of the protein during the simulation, suggesting the inhibitory potential of **CSMS00081585868** against *Pf* 5-ALAS.

The limitation of this study is that little is still known about the biology of *Plasmodium falciparum*, especially the liver stage cycle of the parasite (42, 43). However, the advent of computer-aided drug design (CADD) has made it possible to predict the activities of several targets in the parasite and thereby design potential inhibitors against the targets. In conclusion, the essentiality of the heme biosynthetic pathway of *Pf* at the liver stage presents an opportunity to design prophylactic drugs to reduce the incidence and burden of malaria disease in endemic regions. In this study, following *Pf* 5-ALAS model design, target evaluation, macromolecular processing, library preparations of 2,621 compounds and processing, we employed structure-based virtual screening to identify potential lead compounds possessing higher inhibitory and drug-like properties to *Pf* 5-ALAS than the cofactor pyridoxal 5′-phosphate. We recommend that hit-to-lead (H2L) optimization should be employed, with the idea of the qualitative structural assessments, to improve the drug-like efficiency of the identified hits.

## Data availability statement

The original contributions presented in this study are included in this article/supplementary material, further inquiries can be directed to the corresponding author.

## Author contributions

EA, GO, RA, and IA spearheaded the project and collaborated with AV to design the work. GO, RA, and IA interpreted the results and wrote the first draft of the manuscript. All authors reviewed and approved the final draft.

## Footnotes

1
http://pharmit.csb.pitt.edu/
